# The current three-year postgraduate program in urology is insufficient to train a urologist

**DOI:** 10.4103/0970-1591.42614

**Published:** 2008

**Authors:** Gagan Gautam

**Affiliations:** Department of Urology and Renal Transplant, Fortis Flt. Lt. Rajan Dhall Hospital, Vasant Kunj, New Delhi - 110 070, India

“We must all obey the great law of change. It is the most powerful law of nature.”
- **Edmund Burke**

The future of any profession depends almost entirely on the capability and vision of its youngest members. In case of urology, it is the residents who will determine the shape of this dynamic field well into this new century and beyond. It is imperative therefore, to not only ensure that the best and the brightest choose to join our fold but also to train them well so that our medical system can keep pace with the changing urological scenario.

## THE UROLOGICAL REVOLUTION

In India, we are currently at the doorstep of a urological revolution. The advent and growth of new subspecialties has resulted in a rapid explosion of urological knowledge and skills.[1] This has led to a trend whereby urologists tend to become trained experts in a particular subspecialty while ignoring the other aspects of a general urological practice. While this may be beneficial in selected institutions, most urologists in peripheral centers who cater to the majority of the Indian population need to be well versed in general urological skills covering a wide array of these new subspecialties. Clearly, in India, there is no escaping the need to have a core competency in the management of common urological diseases.

The original Indian residency program was designed at the time when urology was quite a limited specialty. There was no endourology, no laparoscopy, no lithotripsy, and no robotics. Reconstructive and pediatric urology were in their infancy. Microsurgery for male infertility was not developed. Urology at that time essentially involved open surgery for stone and malignancy. With the emergence of new modalities of treatment, there is also a greater amount of time required to train the budding urologists in these skills. The current three-year urology residency may not provide the right foundation for this in these changing times.

## GENERAL SURGERY: ARE THREE YEARS TOO LONG?

There is no doubt that a solid foundation in general surgery is essential for specialization in any surgical branch-but are three years necessary for it? One would think not. An intensive one to two-year general surgery rotation with more time available later on for urology would be good enough to develop the basics and then build on it to make a comprehensively trained general urological surgeon. In the United States in 1991, a third of the urology residency programs (41 of 126) were of five years duration including a two-year pre-urological general surgery training. Over the next 12 years there has been an overwhelming trend of conversion to a one-year general surgery training followed by a four-year urology stint. In fact, in 2003 all US urology programs but one required a four-year clinical urology training.[2]

According to a European survey, residency training in urology proceeds after graduation from medical school in 44% of the European countries, after a compulsory rotatory internship in 26%, after a short duration pre-residency urology ‘elective’ in 19% and after a common general surgery training in only 11%. The only European countries requiring a ‘common trunk’ general surgery residency are Finland, France, Greece and the UK. In all others general surgery training is incorporated within the framework of a urology residency program. In fact, in three countries, namely, Estonia, Italy and Ukraine general surgery training is foregone altogether in favor of a purely urological training program. The mean time spent in general surgery training in European countries is 16.2 months.[3] So if one compares the duration of general surgical training for urologists in India (three years) with that in the US (one year) and Europe (1.3 years), it does seem that our time allocation between general surgery and urology training needs to be redefined.

## DEFICIENCY OF OPERATING EXPOSURE

A large number of residents, even in leading institutions of our country rue a deficiency of practical operating experience in our urology residency programs. This lack of proper practical training is distressing - although not uniquely Indian. In a survey of laparoscopic training during residency, Duchene *et al.*, discovered that only 38% of US urology residents felt that their laparoscopic experience was at least average or acceptable.[4] Another recent study ‘despairingly’ concludes that an average US resident gets to perform a mean of ‘only’ 6.7 and 9.5 continent and incontinent urinary diversions respectively, during the course of his/her training and calls for corrective measures in this regard.[5] Similar surveys have never been done in India. Nevertheless, it is likely that Indian data on this aspect of our residency programs would present an alarming picture.

Most of our institutions still lack a structured policy regarding the number and type of surgical procedures to be performed by residents in training. Surgeries are allotted to residents in a relatively random fashion and the most important factor in this is the amount of trust and rapport a resident has been able to develop with the members of the faculty. Although this is not necessarily improper, it does introduce a discrepancy in the surgical exposure between two residents in the same or different departments. In our current residency system, by the time a resident comes into a situation where he would start getting some surgical freedom, it is time to go. By increasing the number of years to be spent in the same department, the independent surgical exposure would definitely be enhanced and would go a long way in producing urologists who are more technically skilled than those coming out of residency programs today.

## RESEARCH: THE NEGLECTED COMPONENT

Clinical (and even basic science) research is a vital component of a residency training program. Not only does it enrich the individual during training and thereafter, but also ensures growth and development of our specialty in the future.[6] It would be a personal experience of many teachers that the best residents are usually also the best researchers and usually have more publications and presentations than their counterparts. In the current three-year program, residents are often left performing a desperate balancing act between inpatient care, operating room duties, emergency calls and preparation for examinations leaving very little room and I dare say, inclination for research. This is not only detrimental for the individual but also for the institution since the reputation of the institution and its stature in scientific circles is determined to a large extent by the publications emerging from it.

According to a Medline search performed on 15 March 2008, there were a total of 82 Indian manuscripts published in The Journal of Urology, BJU International and Urology (The Gold Journal) during the past three years. This is indeed quite paltry as compared to the 3275 publications from institutions in the United States in these journals during the same time period. Though the reasons for this vast gap may be many, a general lack of time and facilities for urological research in India is definitely quite glaring. A six-year program with a dedicated research year would provide a greater opportunity to perform high quality research during the training period by ensuring a more rational time allocation to this aspect of residency training thereby resulting in an increase in the number of urological publications coming out of Indian academic institutions.

## UROLOGY CURRICULUM IN MBBS

I would concede that the current MBBS curriculum does not offer enough exposure to subspecialties like urology and that makes it difficult for medical students to choose a path that would lead to a direct five to six-year course after MBBS. This doesn't however mean that the current three-year course in urology is adequate. It stresses on the fact that there is a need to change the way medical education is conducted at the undergraduate level. Similar concerns have been voiced in the US with a recent survey showing that only 17% of US medical schools had a compulsory one to two-week urology rotation during undergraduate medical training.[7] In the latest study on this topic, 65% of the program directors felt that it was possible for a US medical student to graduate without having had any undergraduate exposure to urology.[8] On the other hand it has been shown in a recent French study that a practical exposure to urology during the early part of medical training ensures a greater appeal and preference for urology and is reflected in an increase in the number of medical students choosing this subject for specialized training.[9] These factors notwithstanding, urology continues to be one of the most competitive residency programs in the US, Europe and indeed in India. In order to assist the medical student in making a decision regarding the specialty to pursue after graduation, a structured curriculum needs to be formulated and implemented at the undergraduate level so as to ensure adequate exposure to subspecialties like urology.

## THE STRESS OF UNCERTAINTY

The present portal of entry into a urological residency in India is fraught with uncertainty. After MBBS, at every stage, the prospect of a daunting entrance examination looms ahead. For an aspiring urologist, completion of a general surgery residency is just a means to an end. He/she then has to sit for highly competitive urology entrance examinations, which more often than not result in wasted years and broken hearts. Even these examinations are not standardized. While some institutions ask pure urology, others want to test the candidates on their general surgery knowledge. Still others even put questions from biochemistry and gynecology in their entrance examinations. The candidates end up either riding two horses at the same time (with a high chance of falling) or have to forego some examinations altogether. Clearly, such a system causes an undue amount of stress and anxiety in the minds of these young surgeons and is counterproductive to our medical system. A clear-cut direct six-year course after MBBS will resolve much of these apprehensions and would allow the students to focus on their residency training rather than worry about an entrance examination at every step. Moreover, it will also be helpful to the institutions since it would obviate the need to hold an entrance examination every year or six months to induct new urology trainees into their departments.

In spite of having developed an interest in a surgical field during MBBS, many young medical graduates prefer disciplines like radiodiagnosis and dermatology to general surgery since they are wary and uncertain about their prospects of obtaining a residency position in a surgical subspecialty of their choice. In many of these cases they cannot be faulted for choosing safety over uncertainty. It is possible that our subspecialty i.e. urology may be losing a phenomenal amount of promising talent to other specialties and subspecialties in this way. In the interest of our future, it is imperative that we attract this “cream” to make our profession richer.

## CONCLUSIONS AND RECOMMENDATIONS

So what are the changes that can potentially help us in establishing world-class urological training programs in India? I propose certain suggestions, which I hope, will stimulate a discussion in this regard. The main objectives behind these suggestions are to attract the best medical students into urology, to ensure a level playing field for all candidates while entering or exiting a urology residency program, to allocate the right amount of time between general surgery and urology and to establish a similar standard of training between all urology training programs in the country.

1. Urology to be converted to a broad specialty with a six-year training program.
2. Admissions to this specialty to be made after a competitive entrance examination after MBBS.
3. The All India PG entrance examination can be the common pathway to all urology seats in the country whereby students can choose their institutions during counseling according to merit.
4. Initial one year of the residency to be spent in the general surgery department. At the end of one year the students would need to qualify in an MCQ + Viva Voce examination in general surgery prior to moving to the urology department for the next five years of continuous urology training.
5. The urology component to have a compulsory research year for research in basic science/urology. Uniform criteria with regards to the number of publications during the research year to be established.
6. A supervising body (possibly the USI) should have administrative control over all urology training programs in the country. This body would lay down the format and requirements of the training program in a uniform fashion.
7. A defined number of required supervised surgical procedures in each category to be laid down by this body. Log book maintenance to be made compulsory. It should be compulsory for institutions to ensure that residents get to perform the stipulated number of surgical procedures prior to the exit examination.
8. A common national level exit examination for all programs prior to certification from this body.
9. A limited number of fellowship positions in subspecialties in certain centers of excellence to be established, entry into which will involve an analysis of the publications in that particular subspecialty during the training years and the performance in the national residency exit examination.

Till now, India and the Indian medical system have produced some of the finest surgeons and physicians in the profession. Indian urologists have scripted success stories in almost all rapidly emerging subspecialties of urology. However, at present there is an overwhelming need to change our training patterns with the changing times. We must anticipate the future and rise to the challenges coming our way lest we get left behind in the race for urological expertise…and produce inadequately trained substandard urologists who will not be able to hold their own in the face of the changing urological scenario.
